# Incidences and clinical outcomes of acute kidney injury in ICU: a prospective observational study in Sri Lanka

**DOI:** 10.1186/1756-0500-7-305

**Published:** 2014-05-19

**Authors:** Eranga S Wijewickrama, Gowri M Ratnayake, Chaminda Wikramaratne, Rezvi Sheriff, Senaka Rajapakse

**Affiliations:** 1 Department of Clinical Medicine, Faculty of Medicine, University of Colombo, Colombo, Sri Lanka; 2 Colombo South Teaching Hospital, Kalubowila, Sri Lanka; 3 Medical Intensive Care Unit, National Hospital, Colombo, Sri Lanka

**Keywords:** Acute kidney injury, Intensive care, Critical care, Sepsis, Mortality, ICU stay

## Abstract

**Background:**

Acute kidney injury (AKI) is a common and a serious complication among patients admitted to intensive care units (ICUs), and has been the focus of many studies leading to recent advances in diagnosis and classification. The incidence and outcome of AKI in Sri Lankan ICUs is largely unknown. The aim of this study was to describe the incidence, severity and outcome of AKI among patients admitted to the medical ICU, National Hospital, Colombo, Sri Lanka (NHSL).

**Methods:**

Patients admitted to the medical ICU, NHSL, over a period of 6 months were studied prospectively.

Standard demographic, physiological and clinical data were collected. Severity of illness was assessed using SOFA (Sequential Organ Failure Assessment) score. Diagnosis of AKI was based on Acute Kidney Injury Network (AKIN) criteria.

**Results:**

Of 212 patients screened, 108 satisfied the inclusion criteria; males 67(61.5%); mean age 47.8 years(SD 19.4, range 12-94). Mean duration of ICU stay was 11.6 days (SD 10.6, range 2-55). Eighty one (75.0%) received mechanical ventilation. Forty nine (45.4%) had sepsis. ICU mortality was 38.9% and AKI was present in 60.2%. The majority of AKI patients (38, 58.5%) had AKI stage 3. Patients with AKI were at higher risk of death (p < 0.01). Neither age, gender, nor the presence of co-morbidities were associated with increased risk of AKI. Patients with AKI had significantly longer ICU stay (Log-Rank Chi Square: 23.186, p < 0.0001). Both the incidence of AKI and ICU mortality were higher in patients with SOFA scores over 9 (Pearson Chi-Square 7.581, p = 0.006, and 11.288, p = 0.001 respectively).

**Conclusions:**

The incidence of AKI is high at 60% among our ICU patients, and those with AKI had higher mortality and longer duration of ICU stay. Age, gender or the presence of co-morbidities was not associated with a higher risk of AKI. Patients with SOFA scores over 9 within the first 24 hours were more likely to develop AKI and had higher risk of death.

## Background

Acute kidney injury (AKI) is a common and major complication in critical care, and is associated with poor outcome [1]. The Acute Kidney Injury Network (AKIN) criteria are currently used to stage acute kidney injury in practice [2], although RIFLE (Risk, Injury, Failure, Loss & End-stage kidney disease) criteria [3] have been used in most large epidemiological studies. Crude mortality has been shown to be higher with worsening stages of AKI [4]. Data on the aetiology, management practices and outcome in patients admitted to intensive care units in Sri Lanka has not been studied in depth. The aetiology and patterns of AKI, as well as management practices are likely to differ in countries with resource limitations and variations in disease patterns. Tropical infectious diseases are an important cause of admission to ICU in Sri Lanka, and certain tropical diseases such as leptospirosis have a high propensity to cause AKI. Continuous renal replacement therapy is not routinely available in many ICUs in Sri Lanka [5].

The Medical Intensive Care Unit of the National Hospital, Colombo, Sri Lanka, is a dedicated 9 bedded medical ICU in the largest hospital in Sri Lanka. This ICU has access to intermittent haemodialysis but has no continuous renal replacement facilities. Haemodynamically unstable patients receive conventional peritoneal dialysis where required. Admissions are based on priority need, and are received largely through direct admissions to ICU from the emergency treatment unit, and from the medical wards of the hospital. The case-mix is wide, but comprises only patients primarily with medical problems. Specialist cover is provided mainly by general internal medicine consultants, with anaesthetic consultants providing input for vital organ support. The unit is staffed 24 hours by middle grade (non-specialist, non-training grade) doctors with special training in critical care, nurses with critical care training, physiotherapists and other support staff.

The aims of this study were to: a) to describe the frequency, patterns, aetiological factors, and risk factors, leading to AKI; b) to determine the relationship between AKI and outcome; c) to determine the relationship between the SOFA score and the likelihood of developing AKI and mortality.

## Methods

This was a prospective observational study in the medical intensive care unit (MICU) of the National Hospital, Colombo, Sri Lanka over a six-month period. We screened all patients admitted to the MICU for inclusion. We excluded patients who had chronic kidney disease and were on renal replacement therapy, and those whose duration of ICU stay was shorter than 48 hours.

Standard demographic, clinical and physiological data was obtained prospectively from all the patients.

Demographic information included age, gender and date of admission. Clinical data included the primary diagnosis, presence of co-morbidities and the need for mechanical ventilation. Physiological data included Glasgow Coma Scale, arterial oxygen tension (PaO_2_)/fraction of inspired oxygen (FiO_2_) ratio, blood pH, serum sodium, potassium, bilirubin, haemoglobin, platelet and white cell count. Data on kidney function included serum creatinine, urea and urine output. Severity of illness on admission and during the ICU stay was assessed using the SOFA score.

We defined several primary diagnostic categories based on the primary reason for admission to ICU; classification into a diagnostic category was based on the diagnosis documented at the point of admission by the treating clinician. A diagnosis of sepsis/septic shock was made where the primary reason for admission was a sepsis related diagnosis [6], and included sepsis associated with pneumonia, gastrointestinal disease, urinary tract infections, central nervous system infections, soft tissue infections and sepsis of undetermined source. A cardiac diagnosis was made where the primary reason for admission was cardiogenic shock (systolic blood pressure <90 mmHg, absence of hypovolemia, and clinical signs of poor tissue perfusion i.e., oliguria, cyanosis, cool extremities, altered mentation [7]), cardiac arrest, congestive cardiac failure (bilateral basal crackles, cardiomegaly, elevated jugular venous pressure [8]) and acute myocardial infarction (rise in troponin and either ischaemic chest pain, new ST-T wave changes or pathological Q waves on ECG [9]). A respiratory diagnosis encompassed primary respiratory arrest, aspiration syndrome, non-cardiogenic pulmonary oedema (not related to sepsis), exacerbations of chronic obstructive pulmonary disease or asthma, and pulmonary embolism. A diagnosis of gastrointestinal haemorrhage included bleeding due to peptic ulcers, diverticulosis and varices. All other non-surgical gastrointestinal diagnoses were categorized as ‘other’.

A metabolic/poisoning diagnoses included non-operative causes of metabolic coma, diabetic ketoacidosis, drug overdose or other endocrinopathies. Primary neurologic diagnoses included stroke, intra-cerebral haemorrhage, subarachnoid haemorrhage, epidural haematoma or other neurological causes for coma.

AKI was defined based on the AKIN criteria [2], as an abrupt (within 48 hours) reduction in kidney function (absolute increase in serum creatinine of ≥0.3 mg/dl (≥26.4 μmol/l), percentage increase in serum creatinine of ≥50% (1.5-fold from baseline), or reduction in urine output (documented oliguria of <0.5 ml/kg per hour for more than six hours)). The 3 stages of AKI were considered to be: stage 1- increase in serum creatinine of ≥0.3 mg/dl (≥26.4 μmol/l) or increase to ≥150% to 200% (1.5-2-fold) from baseline or urine output < 0.5 ml/kg per hour for >6 hours; stage 2- increase in serum creatinine to >200% to 300% (>2- to 3-fold) from baseline or urine output <0.5 ml/kg per hour for >12 hours; stage 3- increase in serum creatinine to >300% (>3-fold) from baseline (or serum creatinine of ≥4.0 mg/dl (≥354 μmol/l) with an acute increase of at least 0.5 mg/dl (44 μmol/l) or urine output <0.3 ml/kg per hour for 24 hours or anuria for 12 hours.

Data was collected using a structured datasheet, administered by a research assistant, from the case notes and by interviewing the patients (or relatives where the patients were unable to communicate) and treating physicians. Collected data were analyzed using SPSS version 16.0®. Categorical data were expressed as proportions and subgroups were analyzed using Pearson Chi-square test. Kaplan-Meier analysis was used to compare length of stay.

Ethics clearance was obtained from the Ethics Review Committee, National Hospital, Colombo on 07^th^ December 2011 (No AA/ETH/2011). Informed written consent was obtained from patients, or from the closest relative where the patient was too ill to communicate. All investigations that the patients were subjected to were a part of the routine workup done in any critically ill patient. No personal information was collected, and individual patient data was coded to the bed-head ticket number. All datasheets were kept in a locked cabinet by the senior investigators.

## Results

**Figure 1:**
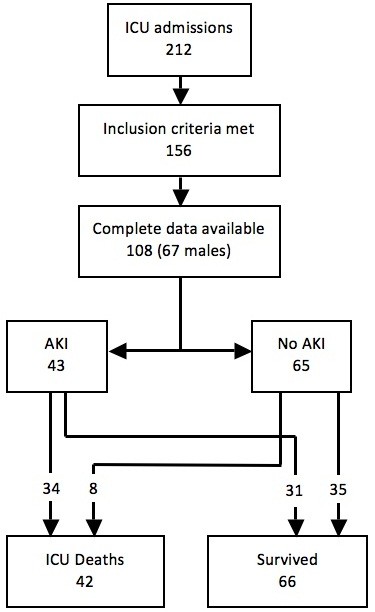
Flowchart of patients recruited to study.

**Figure 2:**
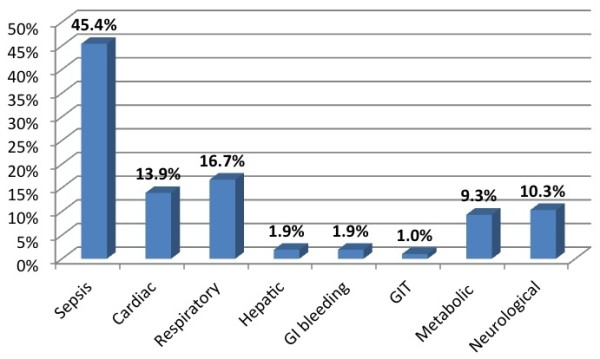
Frequency of primary clinical diagnoses of patients.

**Figure 3:**
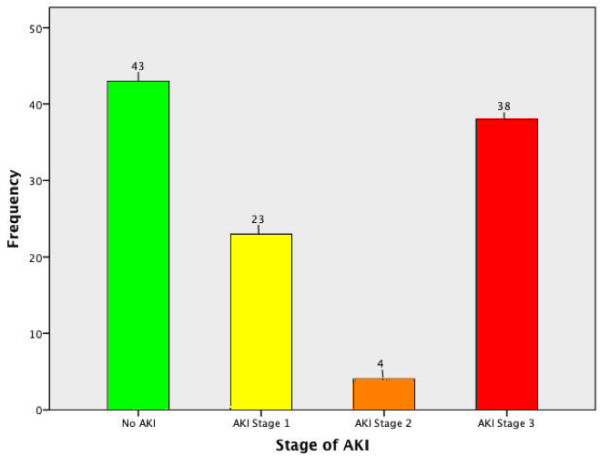
Frequency of AKI in ICU patients.

**Figure 4:**
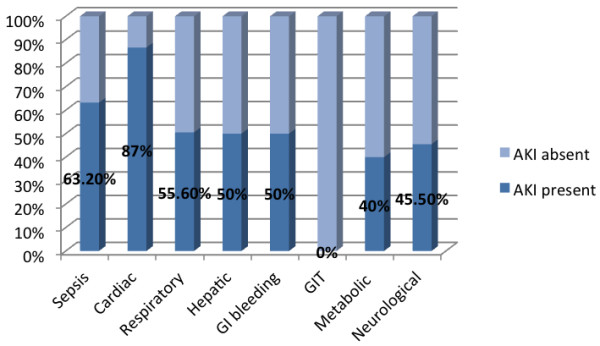
Incidence of AKI in relation to primary diagnosis.

A total of 212 patients admitted to the MICU, NHSL during the study period were screened for inclusion into the study. Of these, 156 (73.6%) patients satisfied the inclusion criteria; complete data was available in 108 (69.2%), and these patients were included in the final analysis (Figure 1). The majority (67, 61.5%) were males. The mean age was 47.8 years, SD 19.4, range 12-94 years. The mean duration of ICU stay was 11.6 days (SD 10.8, range 2 to 55 days). The majority of patients admitted to ICU, (75.0%) received mechanical ventilation during their ICU stay. Sepsis was the commonest primary diagnosis (45.4%) (Figure 2), with cardiac and respiratory pathologies accounting for 13.9% and 16.7% of ICU admissions respectively.During ICU stay, 65 (60.2%) developed acute kidney injury (AKI). The majority with AKI had AKI stage 3 (38, 58.5%) (Figure 3). Thirty seven (71.1%) of the patients in AKI stage 3 required dialysis. Among those who received dialysis, the majority (36, 97.3%) received intermittent haemodialysis, the remainder receiving peritoneal dialysis. Patients with sepsis had a high likelihood (63.2%) of developing AKI; the highest incidence of AKI was observed in those with cardiac pathologies as the primary diagnosis (87%) (Figure 4).

**Figure 5:**
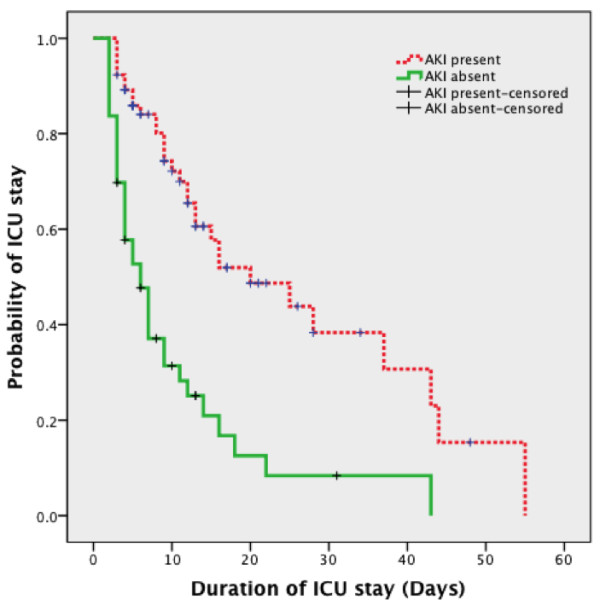
Kaplan-Meier plot comparing duration of ICU stay among patients with and without AKI on admission.

Just under half of the patients (49, 45.4%) had medical co-morbidities. Of the co-morbidities, hypertension was the commonest (28, 57.1%), with diabetes and ischaemic heart disease present in 27 (55.1%) and 17 (34.7%) respectively. Age, gender, and the presence of co-morbid illnesses such as diabetes mellitus, hypertension or ischaemic heart disease were not associated with developing AKI (p > 0.05) (Table 1). The duration of ICU stay was significantly longer among patients with AKI compared with no AKI (Kaplan Meier analysis, Mantel-Cox Log Rank Chi Square: 23.186, p < 0.0001) (Figure 5).

**Table 1:** Association of AKI with potential risk factors

**Risk factor**	**AKI**	**No AKI**	**Total**	P-value*
Diabetes mellitus				
Yes	19 (70.4%)	8 (29.3%)	27 (100%)	0.26
No	46 (56.7%)	35 (53.3%)	81 (100%)	
Hypertension				
Yes	20 (64.7%)	8 (35.7%)	28 (100%)	0.18
No	45 (56.3%)	35 (44.7%)	80 (100%)	
Ischaemic heart disease				
Yes	11 (64.7%)	6 (35.7%)	17 (100%)	0.79
No	54 (59.3%)	37 (40.7%)	91 (100%)	
Gender				
Male	40 (58.8%)	28 (41.1%)	68(100%)	0.83
Female	25 (62.5%)	15 (37.5%)	40 (100%)	
Age				
≤ 60 years	42 (55.3%)	34 (44.7%)	76 (100%)	0.13
>60 years	23 (71.9%)	9 (29.1%)	32 (100%)	

*Fisher’s exact test.

The ICU mortality in the population was 38.9%. Of those who had AKI only 47.7% were alive on discharge from the ICU, compared with 76.8% of patients without AKI (Table 2). There was a statistically significant association between the presence of AKI and ICU mortality (OR: 4.798, 95% CI: 1.933-11.912) (Table 2).

**Table 2:** Mortality in ICU in relation to the presence of absence of AKI

	**ICU survival**		**Total**
	**Dead**	**Alive**	
AKI absent	**8**	**35**	**43**
AKI present	**34**	**31**	**65**
• Stage 1	13	10	23
• Stage 2	2	2	4
• Stage 3	19	19	38
Total	**42**	**66**	**108**

(Patients with AKI were more likely to die in ICU (Pearson Chi-square:12.369, p < 0.0001)).

AKI stages: stage 1: increase in serum creatinine of ≥0.3 mg/dl (≥26.4 μmol/l) or increase to ≥150% to 200% (1.5-2-fold) from baseline or urine output < 0.5 ml/kg per hour for >6 hours; stage 2: increase in serum creatinine to >200% to 300% (>2- to 3-fold) from baseline or urine output <0.5 ml/kg per hour for >12 hours; stage 3: increase in serum creatinine to >300% (>3-fold) from baseline (or serum creatinine of ≥4.0 mg/dl (≥354 μmol/l) with an acute increase of at least 0.5 mg/dl (44 μmol/l) or urine output <0.3 ml/kg per hour for 24 hours or anuria for 12 hours.

The SOFA score within the first 24 hours of admission to ICU ranged from 1 to 17. A SOFA score greater than 9 within the first 24 hours of ICU admission was seen in a total of 49 patients (45.3%). ICU mortality was significantly higher among patients with SOFA scores over 9 (Pearson Chi-square 7.581, p = 0.006) (Table 3). Similarly, AKI was significantly more likely to occur in patients with SOFA scores greater than 9 (Pearson Chi-square 11.288, p = 0.001).

**Table 3:** SOFA scores in relation to the incidence of AKI and mortality

	**SOFA score**		P-value*
	**= < 9 (n = 59)**	**>9 (n = 49)**	
AKI (%)	27 (45.7%)	38 (77.5%)	0.001
ICU mortality (%)	16 (27.1%)	26 (53.1%)	0.006

(Both AKI and mortality were higher in patients with SOFA scores over 9).

*Pearson Chi-square.

## Discussion

AKI occurring in ICU is a dangerous complication, irrespective of its aetiology. Our findings show a significantly higher risk of death associated with the development of AKI. Whether this increased risk of death is due to complications resulting from AKI per se cannot be determined from this study. Similarly, AKI is associated with significantly longer ICU stay, resulting in a significant burden in terms of cost of care. This is of particular importance in a resource limited setting, as ICU beds are limited and in great demand. Our findings are similar to studies in other countries [1,10] and our results are the first published data on the subject in an ICU in Sri Lanka. A similar but larger study in Italy showed that around half the patients admitted to ICU showed some form of AKI, and 65% of patients in ICU developed AKI at some stage during their stay, a figure very close to that seen in our study [10].

The incidence of AKI has increased gradually over the recent years [11], possibly due to an increase in the use of aggressive diagnostic and therapeutic interventions (in particular contrast imaging) [12]. Mortality due to AKI has shown some evidence of decline over the years, especially in certain subgroups such as trauma, haematological malignancy and cardiovascular surgery [1], although it remains one of the most important causes of death in ICU patients.

As expected, sicker patients were more likely to develop AKI; those with SOFA scores greater than >9 were more likely to develop AKI, and, as expected, had higher mortality. Sepsis and cardiovascular causes resulted in a high incidence of AKI, and older age was also an important risk factor. In our study we found no correlation between age, gender or any of the co-morbidities and the incidence of AKI; in contrast other studies have shown AKI to be associated with older age, male gender and cardiovascular disease and hypertension [10]. The reason for this difference is unclear, but could potentially be due to the higher incidence of sepsis in our cohort and the fact that over 75% of our patients belonged to a younger age group.

No specific preventive strategies are available for AKI apart from adequate fluid resuscitation and avoidance of nephrotoxic agents, in particular intravenous contrast [13,14]. Inotropic support to augment blood pressure is of benefit, and noradrenaline is the preferred agent; low dose dopamine is no longer thought to be useful [6]. Other agents which showed promise in the past, such as tight glycaemic control, corticosteroids, and activated protein C, have failed to show therapeutic benefit in preventing AKI or improving mortality [6]. Dialysis is certainly beneficial, although the exact dose of dialysis remains controversial [15]. While overall there is no evidence that continuous renal replacement therapy is superior to conventional haemodialysis, most studies evaluating this have excluded patients who are haemodynamically too unstable to tolerate conventional haemodialysis [16]. The ICU where this study was performed does not have routine access to continuous renal replacement, thus most patients are managed with intermittent haemodialysis; this makes management of AKI in the setting of shock difficult. Peritoneal dialysis is sometimes used in such situations, although evidence suggests that it is inferior to haemodialysis [17]. Haemodialysis is costly, and is a significant burden on the healthcare system, especially in developing countries.

## Conclusions

The incidence of AKI is high in patients admitted to ICU, and the development of AKI is associated with poor outcome and reduced survival. AKI significantly increases the duration of ICU stay, and this is likely to add to the healthcare burden. Age, gender or the presence of comorbidities do not appear to influence the incidence of AKI in our ICU patients. However sicker patients, with higher SOFA scores, are more likely to develop AKI, and are less likely to survive.

### Ethical approval

Ethics Review Committee, National Hospital, Colombo, Sri Lanka.

## Competing interests

The authors declare that they have no competing interest.

## Authors’ contributions

SR, EW & CW conceptualized the study and developed the initial study protocol. RS reviewed the study protocol and provided further academic input. CW and GR collected data. GR, EW and SR analyzed the data and wrote the first draft of the paper. All authors contributed to the writing of the paper and reviewed and approved the final manuscript.
